# Multisystem Inflammatory Syndrome in Children, Chile, May–August 2020

**DOI:** 10.3201/eid2705.204591

**Published:** 2021-05

**Authors:** Carmen Niño-Taravilla, Hugo Otaola-Arca, Natalie Lara-Aguilera, Yuri Zuleta-Morales, Paula Ortiz-Fritz

**Affiliations:** Clinica INDISA, Santiago, Chile (C. Niño-Taravilla);; Hospital Roberto del Río, Santiago (C. Niño-Taravilla, N. Lara-Aguilera, Y. Zuleta-Morales, P. Ortiz-Fritz);; Clinica Alemana de Santiago, Santiago (H. Otaola-Arca)

**Keywords:** coronavirus disease 2019, children, Kawasaki disease, pediatric intensive care units, multisystem inflammatory syndrome in children, MIS-C, shock, coronary dilatations, COVID-19, SARS-CoV-2, respiratory infections, severe acute respiratory syndrome coronavirus 2, coronavirus disease, zoonoses, viruses, coronaviruses, Chile

## Abstract

We describe 26 children with multisystem inflammatory syndrome associated with coronavirus disease in the pediatric intensive care unit of Roberto del Río Hospital (Santiago, Chile). In total, 10 (38.5%) children required mechanical ventilation; 13 (50.0%) required inotropic support. In addition, 18 (69.2%) patients had echocardiographic abnormalities. No patients died.

On March 11, 2020, the World Health Organization declared a coronavirus disease (COVID-19) pandemic. Acute respiratory failure is the most common complication of COVID-19 in adults (*1*); as of February 2021, COVID-19 has been associated with 2.4 million deaths according to the World Health Organization (https://www.who.int/publications/m/item/weekly-epidemiological-update---23-february-2021). Most children and adolescents with COVID-19 have mild disease that does not require medical intervention (*2*).

In April 2020, a total of 8 previously healthy children with hyperinflammatory shock in the United Kingdom tested positive for antibodies against severe acute respiratory syndrome coronavirus 2 (SARS-CoV-2), the causative agent of COVID-19 (*3*). Consequently, the Royal College of Pediatrics and Child Health proposed the diagnosis of multisystem inflammatory syndrome associated with COVID-19 in children (MIS-C), defined as a persistent fever, inflammation, and evidence of organ dysfunction, after the exclusion of any other microbial cause, with or without PCR confirmation of SARS-CoV-2 infection (*4*). On May 14, 2020, the US Centers for Disease Control and Prevention issued an advisory for MIS-C; the same day, the World Health Organization also issued a report with a case definition of MIS-C (https://emergency.cdc.gov/han/2020/han00432.asp). Researchers have since reported similar cases in the United States (*5*) and Europe (*6*–*9*). The signs and symptoms of MIS-C can resemble Kawasaki disease, toxic shock syndrome, hemophagocytic lymphohistiocytosis, and macrophage activation syndrome (*10*). 

Few publications on COVID-19 in children (*11*) and MIS-C (*12*) have reviewed cases in Latin America. We describe the clinical characteristics, treatment, and results of a cohort of children admitted to the pediatric intensive care unit (PICU) with MIS-C in a tertiary hospital in Chile.

## The Study

We analyzed patients with MIS-C treated in the PICU of Roberto del Río Hospital (Santiago, Chile) during May 11–August 30, 2020 (Figure). We used the case definition of MIS-C proposed by the Ministry of Public Health of Chile (*13*).

**Figure:**
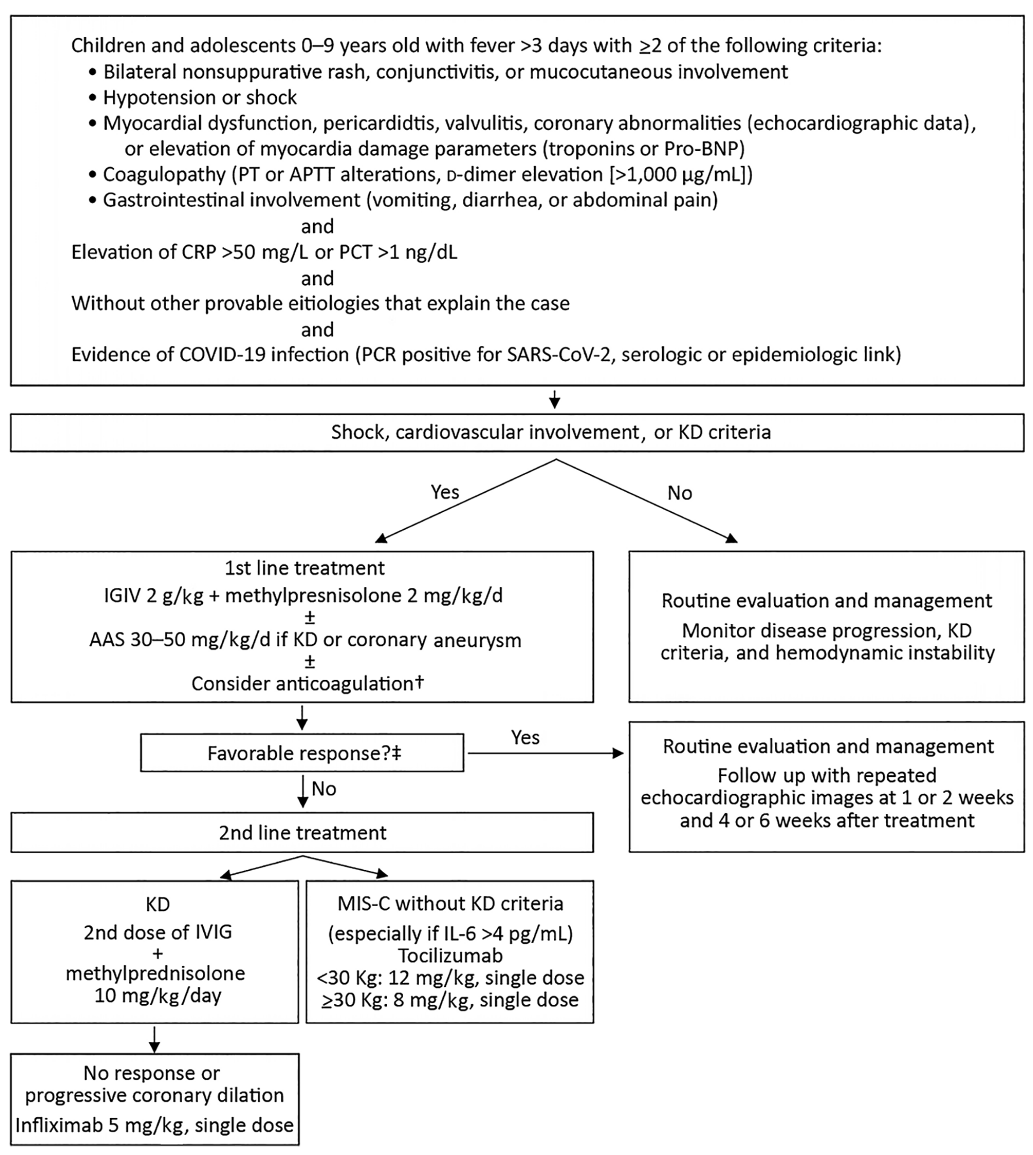
Treatment algorithm for children with multisystem inflammatory syndrome associated with COVID-19, Chile, May–August 2020. †Prophylactic anticoagulation was considered if D-dimer was >1,000 ng/dL or progressively increasing: treatment was 1 mg/kg/d of low molecular weight heparin (Enoxaparin). When thrombosis was suspected or confirmed, the dose was increased to 1 mg/kg every 12 hours and adjusted with anti-Xa factor activity. ‡Favorable response was considered absence of fever for 48 hours, hemodynamic stability, and improvement of inflammatory parameters. AAS, acetylsalicylic acid; APTT, activated partial thromboplastin time; COVID-19, coronavirus disease; CRP, C-reactive protein; IVIG, intravenous immunoglobulin; KD, Kawasaki disease; MIS-C, pediatric inflammatory multisystem syndrome temporally associated with coronavirus disease; PCT, procalcitonin; pro-BNP, pro–brain natriuretic peptide; PT, prothrombin time; SARS-CoV-2, severe acute respiratory syndrome coronavirus 2.

We collected demographic data, medical history, clinical symptoms, and physical examination findings, as well as results of imaging, cardiac, and laboratory tests conducted during the patient’s stay in the emergency room and PICU. We also analyzed data on treatment, complications, outcome, and length of PICU and total hospital stay. The institutional ethics committee of Roberto del Rio Hospital approved the study protocol. We described categorical variables with absolute frequencies and percentages; we described continuous variables with medians and IQRs.

Of the 33 patients with SARS-COV-2 who were hospitalized in the PICU during the study period, 26 met the definition for MIS-C. In total, 16 (61.5%) of these 26 patients met the criteria for Kawasaki disease. The median age was 6.5 years (IQR 2–10.5 years); 15 (57.7%) patients were male. Only 1 patient had a chronic underlying condition (Table 1) (*14*).

**Table 1:** Characteristics of 26 children with multisystem inflammatory syndrome associated with coronavirus disease, Chile, May–August 2020*

Characteristic	Value
Median age, y (IQR)	6.5 (2.0–10.5)
Sex	
M	15 (57.7)
F	11 (42.3)
Concurrent conditions	
No	20 (76.9)
Obesity/overweight	3 (11.5)
Preterm	1 (3.8)
Acute lymphoblastic leukemia, Down syndrome	1 (3.8)
Nationality	
Chilean	19 (73.1)
Venezuelan	3 (11.5)
Peruvian	2 (7.7)
Colombian	1 (3.8)
Haitian	1 (3.8)
Contact with SARS-CoV-2–positive patient	10 (38.5)
SARS-CoV-2 PCR results	
Negative	18 (69.2)
Positive	7 (26.9)
Indeterminate	1 (3.8)
SARS-CoV-2 serologic assay results	
Negative	3 (16.7)
IgM– IgG+	9 (50.0)
IgM+ IgG+	6 (33.3)
Positive PCR results for other co-infections	
Rhinovirus	1 (3.8)
Rhinovirus and adenovirus	1 (3.8)
Signs and symptoms	
Fever	26 (100.0)
Cough	7 (26.9)
Diarrhea	16 (61.5)
Vomiting	12 (46.2)
Abdominal pain	17 (65.4)
Conjunctivitis	15 (57.7)
Rash	16 (61.5)
Shock	24 (92.3)
Vasoplegic	18 (75.0)
Cardiogenic	1 (4.2)
Mixed	5 (20.8)
Myocarditis	12 (46.2)
Treatments	
Invasive mechanical ventilation	10 (38.5)
Vasoactive drugs	13 (50.0)
Hyperimmune plasma	1 (3.8)
Corticoids	23 (88.5)
Intravenous immunoglobulin, doses	
1	20 (76.9)
2	2 (7.7)
Tocilizumab	3 (11.5)
Infliximab	1 (3.8)
Acetylsalicylic acid	19 (73.1)
Low molecular weight heparin	19 (73.1)
Not used	7 (26.9)
Prophylaxis	17 (65.4)
Treatment	2 (7.7)
High-flow hemofiltration	1 (3.8)
Deaths	0
Median intensive care unit admission, d (IQR)	5 (2.0–7.0)
Median hospitalization duration, d (IQR)	10 (7.3–16.8)
Echocardiogram results	
Normal	8 (30.8)
LV systolic dysfunction†	4 (15.4)
Pericardial effusion	3 (11.5)
LV systolic dysfunction and pericardial effusion	3 (11.5)
Hyperdynamic	1 (3.8)
Mild LV diastolic dysfunction‡	2 (7.7)
Coronary alterations§	5 (19.2)

*Values are no. (%), except as indicated. IQR, interquartile range; LV, left ventricle; SARS-CoV-2, severe acute respiratory syndrome coronavirus 2. †Defined as LV ejection <55%. ‡Defined as decreased E wave. §Defined as: Z-score 2–2.5, dilatation; Z-score 2.5–5, small aneurysm; Z-score 5–10, medium aneurysm; Z-score >10; giant aneurysm (*14*).

In total, 22 (84.6%) patients tested positive for SARS-CoV-2 infection, 7 (26.9%) by reverse transcription PCR and 15 (57.6%) by serologic assay. The other 4 (15.3%) patients tested negative for SARS-CoV-2 but had a COVID-19 exposure. The most frequent symptoms were fever (26, 100%), shock (24, 92.3%), abdominal pain (17, 65.4%), diarrhea (16, 61.5%), vomiting (12, 46.2%), rash (16, 61.5%), and conjunctivitis (15, 57.7%) (Table 1). 

We also collected data on laboratory test values (Table 2), critical care interventions, treatments, and outcomes (Table 1). Ten (38.5%) patients required mechanical ventilation for a median duration of 4 days (IQR 2.5–5 days). Only 1 (3.8%) patient met the criteria for acute respiratory distress syndrome; that patient had an oxygenation index of 25. Half (13, 50.0%) of the patients required vasoactive drugs. We used high-flow hemofiltration as salvage therapy for refractory shock in 1 patient. No patients required extracorporeal membrane oxygenation (ECMO). In total, 20 (76.9%) patients received intravenous immunoglobulin; 2 (9.1%) received a second dose. We treated 23 (88.5%) patients with corticosteroids; 1 (3.8%) required a larger dose (Figure). We prescribed immunomodulatory agents for 4 (15.4%) patients: tocilizumab for 3 patients and infliximab for 1. 

**Table 2:** Laboratory test values of 26 children with multisystem inflammatory syndrome associated with coronavirus disease, Chile, May–August 2020*

Test	Median value (IQR)		Reference range
	Emergency department	Intensive care unit	
Leukocytes, mm^3^	10,540.0 (7,400.0–15,900.0)	NA	4,500–11,000
Lymphocytes, mm^3^	1,080.0 (732.5–2,579.5)	560.5 (409.5–943.0)	1,500–4,000
Platelets, mm^3^	175,000.0 (96,000.0–232,000.0)	82,000.0 (40,000.0–111,250.0)	150,000–400,000
Albumin, g/dL	3.1 (2.9–3.4)	2.2 (2.0–2.8)	3.4–5.4
Troponins, ng/mL†	NA	0.1 (0.0–1.8)	<0.034
Creatine phosphokinase, U/L	133.5 (55.5–234.0)	100.0 (162.5–220.5)	32–294
Creatinine kinase-MB, U/L	2.2 (1.1–11.9)	3.3 (1.2–13.4)	<12
C-reactive protein, mg/L	134.0 (94.0–300.5)	198.5 (121.5–302.8)	<5
Procalcitonin, ng/mL†	NA	13.0 (2.39–38.0)	<0.5
Ferritin, ng/mL	206.5 (91.5–368.8)	567.0 (304.5–1000.0)	15–150
Triglycerides, mg/dL	175.5 (103.0–244.5)	205.5 (151.5–316.0)	<75
Lactate dehydrogenase, U/L	288.0 (257.0–357.0)	285.5 (261–328.3)	105–333
Glutamic oxaloacetic transaminase, U/L	45.0 (32.0–66.0)	51.0 (36.5–66.0)	0–40
Fibrinogen, mg/mL	457.0 (375.0–513.0)	447.0 (353.25–509.0)	200–400
D-dimer, ng/mL	1,700.0 (730.0–3,500.0)	2,900.0 (1,670.0–3,950.0)	<500
Creatinine, mg/dL	0.6 (0.4–1.2)	0.8 (0.5–1.5)	Varies‡
Type B natriuretic pro-peptide, pg/mL§	NA	1,749.0 (255.8–4,722.8)	<125
Interleukin 6, g/mL¶	NA	322.0 (95.5–621.8)	<3.4

*Emergency department indicates value at admission; intensive care unit value indicates most severe value. IQR, interquartile range; NA, not available. †Troponins and procalcitonin were only measured in the intensive care unit. ‡Creatine reference ranges: <1 mo, 0.3–1.0 mg/dL; 1–12 mo, 0.2–0.4 mg/dL; 1–12 y, 0.3–0.7 mg/dL; >12 y, 0.5–1.1 mg/dL. §Only determined in 6 patients.  ¶Only determined in 10 patients.

In total, 18 (69.2%) patients had echocardiographic abnormalities (Table 1), including 5 (19.2%) patients who met the criteria for Kawasaki disease with coronary artery abnormalities. The median duration of PICU stay was 5 days (IQR 2–7 days). None of the patients died.

## Conclusions

We describe 26 MIS-C patients in the PICU of Roberto del Río Hospital in Chile. In this hospital, the maximum incidence of MIS-C occurred ≈4 weeks after the peak of COVID-19 cases in adults, as described in the literature (*5*–*9*).

The median age of the cohort in our study was 6.5 years, lower than usually reported for patients with MIS-C (8–9 years) (*5*); 2 patients were neonates. Slightly more than half (61.5%) of patients met criteria for typical or atypical Kawasaki disease.

Nearly all (84.6%) patients had laboratory-confirmed SARS-CoV-2 infection. However, whereas many (57.6%) had antibodies against SARS-CoV-2, only 7 (26.9%) tested positive by PCR. These findings suggest that MIS-C might be caused by a hyperinflammatory response after asymptomatic SARS-CoV-2 infection, rather than direct cell injury from active viral replication. Although the syndrome’s pathophysiology has been correlated with the cytokine storm described in adults with severe COVID-19 (*13*), the mechanisms of MIS-C remain unclear.

We observed clinical manifestations similar to those described internationally (*5*–*9*). In this cohort, the most frequent manifestation was fever with gastrointestinal symptoms (65.4%), in agreement with findings described in the literature (*8*).

Similar to previous reports (*5*–*9*), our results showed almost all patients had cardiovascular involvement: 92% had shock and 50% required vasoactive support. Although Roberto del Río Hospital is a national reference center for ECMO, none of the patients in this cohort required extracorporeal supportive treatment; in contrast, Radia et al. (*15*) found that 4% of patients with MIS-C needed ECMO. This difference might be attributable to early immunotherapy.

Approximately two thirds (69.2%) of patients had echocardiographic abnormalities. The most frequent (26.9%) anomaly was left ventricular dysfunction with or without pericardial effusion. In all affected patients, cardiac function recovered before discharge from the PICU. Only 5 (19.2%) of our patients had coronary abnormalities: 4 had a coronary dilatation (Z-score of ≈2.5–2.8) and 1 had a medium coronary aneurysm (Z-score of 6). The frequency of coronary involvement is also consistent with previous reports (*5*–*9*).

We treated nearly all children with intravenous immunoglobulin (76.9%) or corticosteroids (88.5%). Treatment seemed to improve symptoms and decrease inflammatory responses, similar to findings in Europe and the United States (*5*–*9*). According to our treatment protocol, we administered tocilizumab to 3 (11.7%) children; we administered infliximab to 1 (3.8%) child with a medium coronary aneurysm. The main limitations of this study are small sample size and descriptive, nonrandomized design. 

In conclusion, we described 26 children with MIS-C in Chile. Our findings were similar to those reported in other countries. Most patients had echocardiographic abnormalities, and half required vasoactive drug support. We administered immunomodulatory therapy to most patients. Clinical trials and long-term follow-up are needed to elucidate the mechanisms of various treatments and potential sequelae of this condition.
